# Solvents to Fragments to Drugs: MD Applications in Drug Design

**DOI:** 10.3390/molecules23123269

**Published:** 2018-12-11

**Authors:** Lucas A. Defelipe, Juan Pablo Arcon, Carlos P. Modenutti, Marcelo A. Marti, Adrián G. Turjanski, Xavier Barril

**Affiliations:** 1 Departamento de Química Biológica, Facultad de Ciencias Exactas y Naturales, Universidad de Buenos Aires, Buenos Aires 1428, Argentina; ldefelipe@gmail.com (L.A.D.); juanarcon@gmail.com (J.P.A.); cpmode@gmail.com (C.P.M.); 2 IQUIBICEN/UBA-CONICET, Facultad de Ciencias Exactas y Naturales, Universidad de Buenos Aires, Buenos Aires 1428, Argentina; 3 Catalan Institution for Research and Advanced Studies (ICREA), Passeig Lluís Companys 23, 08010 Barcelona, Spain; 4 Faculty of Pharmacy and Institute of Biomedicine (IBUB), University of Barcelona, Avgda. Diagonal 643, 08028 Barcelona, Spain

**Keywords:** molecular dynamics, cosolvent molecular dynamics, drug design, fragment screening, docking

## Abstract

Simulations of molecular dynamics (MD) are playing an increasingly important role in structure-based drug discovery (SBDD). Here we review the use of MD for proteins in aqueous solvation, organic/aqueous mixed solvents (MDmix) and with small ligands, to the classic SBDD problems: Binding mode and binding free energy predictions. The simulation of proteins in their condensed state reveals solvent structures and preferential interaction sites (hot spots) on the protein surface. The information provided by water and its cosolvents can be used very effectively to understand protein ligand recognition and to improve the predictive capability of well-established methods such as molecular docking. The application of MD simulations to the study of the association of proteins with drug-like compounds is currently only possible for specific cases, as it remains computationally very expensive and labor intensive. MDmix simulations on the other hand, can be used systematically to address some of the common tasks in SBDD. With the advent of new tools and faster computers we expect to see an increase in the application of mixed solvent MD simulations to a plethora of protein targets to identify new drug candidates.

## 1. Introduction 

The first revolution in structural biology, in the early 1990’s, increased the available structural information by 20-fold in a decade, creating a high expectation for computational methods that could turn this information into drug candidates. A large body of methods emerged, and some drugs owe their existence—at least in part—to them [1,2]. But it is obvious that the impact of structure-based drug design (SBDD) has not met these expectations. For instance, out of 66 clinical candidates, published by the Journal of Medicinal Chemistry in the 2016–2017 period, none originated as a virtual screening hit [3]. It is a fact that predicting binding affinities (K_A_ = 1/K_D_ = exp(−ΔG_BIND_/RT)) is terribly difficult, and one of the main compounding factors is the solvent’s effect. Contrary to many expectations, designing a good ligand is not a simple matter of finding a molecule that offers a good shape, or electrostatic and chemical complementarity to its protein target. Binding that occurs in the presence of solvent and related predictions will always fall short if this is not fully accounted for. Accurate predictions will unavoidably consider the protein and the ligand embedded in the solvent as part of a condensed state with a great number of configurational possibilities. Molecular dynamics (MD) is uniquely suited to simulate such systems by identifying true ensembles that can be related to macroscopic observables [4]. Here we will review how MD can be used to understand the behavior of water, the universal biological solvent, in relation to the protein surface, and to accurately predict its molecular association properties. We will then discuss how MD simulations of proteins in water and mixed solvents can be used to identify key interactions on their surface, and how these can be incorporated into computational docking, to identify better drug candidates.

We will start by showing that, far from being empty space, a protein’s binding sites in the unbound state are occupied mainly by water (but also ions and metabolites) that does not behave as a homogeneous solvent. Rather, there are well-defined hydration spots and also regions where water density is much lower than in bulk solvents [5]. This determines binding in ways that were not initially expected. Solvation also affects the bound state and binding pathways, thus the gold standard for computational methods is to recapitulate the binding process of a ligand to its target by means of molecular simulations that consider the solvent explicitly. As the timescale of the binding/unbinding events has an exponential relationship with molecular size [6], observing binding on a ‘computational microscope’ [7] is greatly facilitated when the ligand has only a few atoms, particularly if it can be simulated at high concentrations. In Section 2, we will discuss applications and practical aspects of this approach (termed MD simulations with mixed solvents, or MDmix for short). The use of simple ligands as probes to elucidate interaction preferences of protein binding sites has a long history in SBDD. Except for the crucial difference of including explicit solvation in all the computational procedures, MDmix-type simulations can trace their roots to Goodford’s GRID [8], Karplus’ MCSS [9] or the more recent FTmap [10]. All such methods assume that the behavior of the probe is transferable to bigger molecules. Their documented ability to locate binding hot spots confirm that this is at least partially true. But binding free energy is clearly a non-additive property, [11,12] thus it becomes necessary to consider the actual molecules of interest to obtain quantitative predictions. Once considered a dream, major advances in the field of molecular dynamics (See [13,14,15] and references therein) have finally made it possible to directly simulate the binding and unbinding process of actual ligands to their targets. In Section 3 and Section 4, we will review applications with small ligands (fragments) and actual drugs, respectively. We will conclude by discussing the practical limitations and future perspectives for the application of these methods in drug discovery.

## 2. Solvent Structure as a Predictor of Protein-Ligand Interaction Sites 

Among the most relevant processes underlying the formation of protein-ligand complexes is the associated solvent reorganization at the contact surfaces, particularly that of the protein receptor. Water molecules bound to the ligand binding regions must either be displaced to allow direct protein-ligand contact [16,17,18,19] or be retained, bridging specific protein-ligand interactions, as is sometimes observed [20,21,22,23,24,25]. The thermodynamics of this solvent reorganization process is a key contribution to the complex formation free energy and thus to the ligand binding affinity. Initial attention was paid to the role of tightly bound or ordered waters, as revealed by X-ray structures [26], which after displacement by the incoming ligand, were proposed to contribute favorably to ligand affinity. [21,22]. This observation can be further extended to other proteins even if waters are not resolved in diffraction experiments due to lack of resolution, by the use of molecular dynamics in the explicit solvent. 

Explicit solvent molecular dynamics allows studying the structure and dynamics of water molecules, which as a consequence of the shape and charge distribution of protein surfaces, are distributed inhomogeneously in the solvation shell, giving rise to space regions where the probability of finding water molecules is significantly higher (or lower) than that of the bulk solvent, and where rotational and translational motions of each molecule vary significantly. Wiesner et al. for example [27], found that confined waters can have residence times in the range of 1 ns to 106 ns, while for the more mobile waters residence times were only 10–50 ps. Further thermodynamic characterization of these surface waters can be achieved by means of the inhomogeneous fluid solvation theory (IFST), developed by Lazaridis et. al. [5], through the identification of the so-called water sites (WS) [28].

Water sites (sometimes also called hydration sites) are defined as confined space regions close to the protein surface, and internal cavities or packing effects, showing a high probability of finding a water molecule inside them (water finding probability, WFP). They can be evidenced by the presence of crystallographic water molecules, or from MD simulations as defined by their position (whose coordinates correspond to the center of mass of all oxygen atoms, from those water molecules that visit the site during the simulation timescale), their WFP, and their size (characterized by the R90 values, which describes in Angstrom the radius of the WS that contains a water molecule 90% of the time). WS are usually identified by applying a clustering algorithm to a collection of snapshots derived from MD simulations, and despite some special cases, good convergence is achieved in 20–50 ns [29]. 

**Figure 1:**
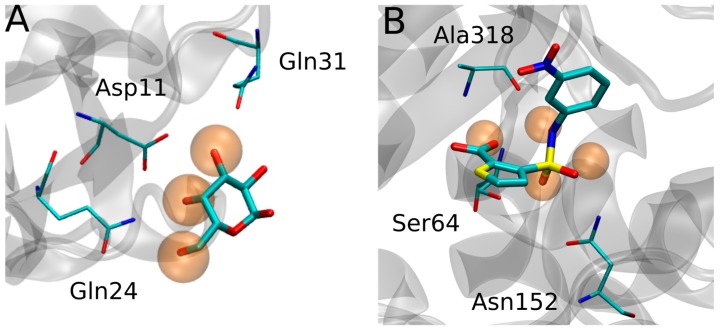
(**A**) Superposition of *Sambucus nigra* agglutinin II in complex with Lactose (PDB ID 3CA4) showing how the Water Sites (Orange transparent spheres) mimic the ligand -OH framework. (**B**) *Escherichia coli* AmpC beta-lactamase (PDB ID 1XGI) WS.

In addition to their application as detailed descriptors of the solvent structure, the relevance of WS determination stems from their capacity to reveal key hydrophilic protein-ligand interaction sites, such as those established by ligand hydroxyl, carbonyl and carboxylate groups, among others. This is nicely exemplified by hydrophilic ligands such as carbohydrates, where several groups reported that the solvent structure in the receptor carbohydrate recognition domain, as revealed by the WS, mimics the framework of the sugar -OH groups, as shown in Figure 1. Moreover, detailed analysis of WS properties showed that those WS that are replaced by the incoming ligand-OH group tend to be those with higher WFP and establishing more interactions with the protein.

More recently, the role WS as predictors of protein-ligand interactions was extended beyond the sugars, again showing that WS, particularly those with high probe finding probability (PFP), tend to be replaced by ligand hydrophilic groups that establish key interactions with the protein receptor, as shown in Figure 1B, for AMPc beta-lactamase. 

Having established the tight relationship between WS and protein-ligand interactions, the next logical move was to apply this knowledge in the context of protein-ligand complex structure prediction (i.e., docking methods) and determination of ligand binding free energies. However, before moving to this topic we will present the use of other solvents as tools for the prediction of protein-ligand interactions. 

## 3. Mixed Solvents Simulations in Drug Design

While water is the universal biological solvent, organic solvents are ubiquitous in laboratories. Some exceptional proteins remain active in neat organic solvents and have been explored as catalysts in industrial applications [30]. More frequently, the buffers used in chemical and structural biology contain small concentrations of organic solvents. Most proteins preserve their structure and function in the presence of 1–5% of DMSO and other common organic molecules [31]. This fact led to the independent observation by NMR and X-ray crystallography that solvents bind preferentially to the active sites of proteins [32,33]. Systematic studies on proteins crystals showed an increasing number of solvent interaction sites as the solvent concentration was increased, and some degree of selectivity for various solvents [34,35]. The most frequently occupied regions coincided with key interaction sites for the substrates, which agreed with the recently postulated notion of ‘hot spots’, i.e., regions on the protein surface that provide most of the binding affinity. [36] Interestingly, the same authors also showed that the computational methods available at the time, GRID [8] and MCSS [9,37] did a mediocre job at predicting binding sites due to the use of implicit solvation and neglecting entropic contributions [34,35]. While the possibility of detecting binding sites by crystallography or NMR with mixed solvents was enticing, the method had limited practical impact because proteins and their crystals rarely withstand high solvent concentrations. Retrospectively, it may seem surprising that it took more than 20 years to perform analogous experiments using molecular dynamics, but it wasn’t until the late 2000’s that MD simulations could routinely explore sufficiently long timescales to ensure meaningful results. In 2009 the Barril’s lab published the first MD application of mixed solvents. In this work, the probe solvent was isopropanol to capture in a single molecule, the hydrophobic and hydrogen bond donor, and acceptor moieties that are common in drug-like molecules. The aim was to detect binding sites and quantify their potential to bind drug-like molecules [38]. This property, often referred to as ‘druggability’ (but note the parallelism with the term ‘ligandability’ [39]), is crucial to predicting the probabilities of successful development of a drug candidate tackling a particular site [40]. The authors noted that “in addition to a prediction for the (druggability of the) whole site, one also obtains a map of the interaction preferences”. Independently and almost simultaneously, the MacKerell’s lab described another mixed solvent approach that focused precisely on this application [41]. In this case, the solvents used were propane as an aliphatic probe, benzene as aromatic probe and water itself was used as a polar probe. Probe interaction maps (called FragMaps) showed an excellent correlation with the binding modes of existing ligands. Since then, a large number of contributions have emerged. Besides druggability [42,43,44] and binding site mapping [45,46], mixed solvents have also been used to predict water displaceability [47,48], to probe protein flexibility and the detection of more druggable conformations [49], or cryptic pockets [50,51,52], or used to re-score docking poses [53,54]. As the diverse implementations and applications of mixed-solvent MD have been extensively reviewed by Ghanakota and Carlson [55], we will place emphasis on the issue of convergence, which is essential for correct predictions.

Convergence of a mixed solvents MD is determined by three interrelated aspects that merit individual discussion: Simulation time, solvent concentration, and protein flexibility.

**Figure 2:**
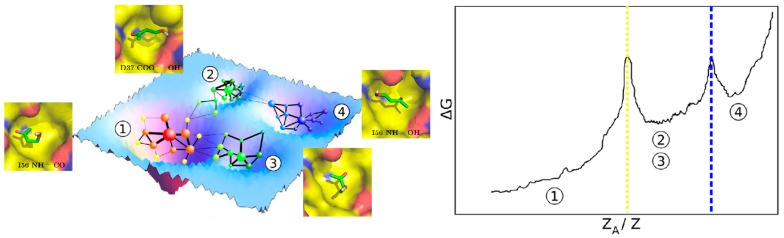
Exhaustive sampling of solvent-protein configurational space enables quantification of binding free energies. The figure is taken from [58].

(1) Simulation time should be sufficient to observe multiple binding and unbinding events. Naturally, the accuracy of the predictions increases and variability decreases as the number of observations (N) increases. Ns as low as 5 are sufficient for qualitative applications but must reach hundreds to be truly quantitative [6]. The other factor determining the total simulation time is the residence time of the solvent (t_1/2_ = ln 2/k_off_; k_off_∝exp(−ΔG^⧧^/RT) [56]. For barrierless dissociation (ΔG_BIND_ = −ΔG^TS^) t_1/2_ depends on the binding free energy, which can increase almost linearly with the number of atoms [57]. Thus, simulation times should increase exponentially with the size of the solvent. But the pathways leading to and from particular binding sites may be hindered, particularly for large ligands, resulting in ΔG_BIND_ << −ΔG^TS^ and, in consequence, much larger t_1/2_. Conventional recipes suggest running several replicas of 10–40 ns each, for a total timeframe of 50–100 ns. This is sufficient to ensure qualitative convergence of the published solvents on the surface of the protein. But direct counting of the number of binding/unbinding events or other forms of measuring convergence should always be used (Figure 2).

(2) Solvent concentration increases sampling effectiveness. Not only due to the increase in effective on-rate (i.e., the number of binding events), but also because multiple binding sites can be sampled simultaneously. The behavior of the organic solvent should remain ideal (i.e., as in infinite dilution) to avoid artifacts caused by solvent-solvent interactions in the unbound state (e.g., inhomogeneous dilution and phase separation). Particular solutions to this problem include the introduction of repulsive terms between solvent molecules [41], or the use of amphiphilic molecules that are highly soluble and do not self-aggregate [59]. Additionally, protein dynamics should not be excessively perturbed by the solvent [60]. Considering that most solvents are denaturants at high concentrations, concentrations should be kept relatively low (<5%), as the protein could be artificially constrained, or simulation times could be much shorter than the denaturation time.

(3) Protein flexibility also determines convergence. Ideally, proteins should be allowed complete conformational freedom, but sampling the configurational space of regular proteins requires excessively long timeframes. Not only that, but it also complicates interpretation of results, as many hotspots are conformation-specific and not representative of the whole ensemble [61]. Constraining the mobility of protein atoms, on the other hand, is a straightforward way of increasing convergence. But this can lead to the overestimation of some hot spots and missing others. As a compromise, for many applications, it is useful and correct to use weak restraints that prevent conformational drift but allow sampling of the local conformational space [61]. Contrarily, if the goal is to induce conformational changes in the protein, such as the opening of cryptic pockets, simulations should be extended to the µs scale [50,51,52].

## 4. Small Ligands and Fragment Screening

Midway between solvent-sized and drug-like molecules, we find the so-called fragments. Fragment-based drug discovery initial hits are small molecules (roughly 10 to 20 non-hydrogen atoms) that are then grown and optimized to become standard drugs (30–40 atoms) [62]. Considering the industrial interest and the small size of these molecules, the use of MD as a screening technique raises considerable interest. In this approach, each compound in the virtual screening collection would be considered a probe that would be subjected to long MD simulations in the presence of the target protein. Probes that bind would then be considered fragment hits. 

At present, molecular docking is the tool of choice for virtual fragment screening. Pioneering work by the Shoichet’s Lab in this area led to the conclusion that although virtual fragment screening is adequate, with hit rates of 14.5% [63] and correct pose prediction, it mostly finds low specificity molecules. The effectiveness of this method for screening and de novo design are well documented in the literature [64,65,66,67,68]. Docking is particularly well suited for fragment screening since the molecules used as fragments are small and not very flexible (less than three rotatable bonds). Nevertheless, if the binding site is not known, it can lead to many false positives. Consensus strategies, like the ones used in FTMap [10], have been used to identify new binding sites. However, in shallow interfaces, as seen in many protein-protein interactions (PPI) sites, the lack of proper treatment for the receptor flexibility can be a drawback for these strategies [69]. 

MD is an essential tool to include receptor flexibility and therefore to compute the binding free energy [1,70]. Both Free Energy Perturbation (FEP) and kinetic parameter estimation methods have been used for fragment discovery, while FEP has been successful for rescoring [71,72] as well as predicting absolute free energies (but not routinely due to high computational cost) [73,74,75]. On the other hand, recent works have focused on the determination of the binding kinetics of small molecules and fragments from MD simulations [76,77,78,79]. Many methods rely on an intelligent design of the analysis strategy to predict the kinetic binding parameters k_off_, mainly using Markov state models [80]. Although most of the reports use molecular simulations to characterize the binding kinetics of known fragments/small molecules [81,82,83], there are some reports on fragment-based screening from “first principles” using molecular simulations [84]. The De Fabritiis’ Lab [85] recently presented a proof of concept of fragment-based screening using MD. They screened a library of 129 fragments (6 to 16 heavy atoms) using short simulations (100 ns), applying a bias and analyzing the trajectories with Markov state models (MSMs). Although the authors found promising fragments binding (8 mM) to the receptor surface (CXCL12), the computational expense is still prohibitive (380,000 GPU hours). 

Work at Shaw D.E. Research sets the bar for quantitative prediction for fragment-based drug discovery [6]. They explored the binding thermodynamics and kinetics of 7 molecules of 4 to 10 heavy atoms to FKBP protein. After hundreds of direct observations of binding and unbinding events, they computed the k_on_, k_off_ and binding affinities. They showed a perfect agreement with FEP simulations, demonstrating that when convergence is ensured, direct simulation of the binding equilibrium by molecular dynamics, can be a quantitative tool. Unfortunately, the RMSE of the computed binding free energy with experimental values was 2.1 kcal/mol, which illustrates the challenges that still lie ahead.

There is significant scope for cross-fertilization between mixed solvent MD and fragment-based drug discovery that has not been extensively explored. For instance, fragments often bind to multiple binding sites on the protein surface [86] which could potentially be identified by cosolvent MD. Fragments can also induce opening of new cavities (cryptic pockets). Gervasio’s research on an exciting tool to address this topic, which combines co-solvent MD and advanced sampling (SWISH), helped to discover cryptic pockets [50,51]. Specifically, simulations on NPC2, p38α, LfrR, and hPNMT were performed, and due to the combined nature of SWISH and CoSolvent, MD was able to find all the cryptic pockets. Once in the binding site, the information derived from the cosolvent MD simulations could potentially be used to predict binding modes and affinities, or to guide the fragment evolution process. Work in this area has been done by MacKerell’s Group with the SILCS methodology. They used the information derived from cosolvent MD to derive so-called FragMaps [41]. These grids were used to rank different ligands and to determine the free energy of binding. 

## 5. Molecular Dynamics Simulations of Drugs or Drug-Like Compounds

Molecular Dynamics simulations could be used to study the free energy of binding of a drug or a drug-like molecule (30–40 heavy atoms) to a protein. This would require the sampling of several binding and unbinding events and therefore unbiased MD runs of at least hundreds of microseconds. Direct observation of drugs binding to their target has been an outstanding achievement of MD applications. Unbiased simulations have revealed the binding pathways of dasatinib to Src kinase [87] and alprenolol binding to the β2 adrenergic G protein-coupled receptor [76]. However, due to the long timescale involved in the dissociation of a drug from its target, direct observation of several unbinding events is not possible. Massive short unbiased simulations in conjunction with Markok State analysis has been used to study benzamidine binding to trypsin [80]. On the other hand, biased simulations can be used to study the Potential of Mean Force of drug binding. Cavalli published the first study of its kind, showing that it was possible to discern active from inactive compounds of the beta-hydroxyacyl-ACP dehydratase of *Plasmodium falciparum* using steered MD [88]. Since then, a large variety of biasing potentials have been investigated and applied to the problem [89]. Even so, the problem remains computationally prohibitive. For instance, the study of a single inhibitor of p38 MAP kinase, that is a fragment of Doramapimod (BIRB 796) and dissociates 4 orders of magnitude faster than the parent compound, took 6.8 μs of production simulations and a total CPU time of 2.5 million core-hours [90]. In addition, identification of the reaction coordinate is often a trial and error process that takes considerable human time and is difficult to automatize [89]. Intriguingly the initial steps of the dissociation may already provide useful information [91], but full reconstruction of the process and quantitative binding affinity estimates remain a major challenge that is only applicable to particularly relevant protein-drug pairs.

For higher throughput applications, docking is widely used to predict protein-ligand interactions and has become extremely useful for virtual screening of huge collections of small molecules [92,93,94]. Most popular docking methods show that success rates are highly system-dependent, with an overall good performance for pose prediction with binding free energy errors of 2–3 kcal/mol for small drug-like molecules and in the absence of significant receptor conformational adjustment [95]. However, it is well known that better results can be obtained by adjusting the docking protocol using previous knowledge for a particular system, such as binding sites or crucial molecular interactions [96,97].

The term “biased docking” (or “guided docking”) refers to the use of additional, experimental or in silico, information to influence the outcome of a docking experiment, e.g., the use of chemical information to favor a certain orientation and conformation of a ligand inside the binding site. The source of this information can be either the protein target structure or its known ligands. A protein-derived bias extracts the information directly from the protein surface and its available molecular interactions and generates a chemically complementary representation of the surface with more weight on particularly important residues, e.g., those confirmed to be essential for the activity by point mutations. As we discussed before, the use of probe atoms, functional groups, small molecules (e.g., mixed solvents) or molecular fragments is another approach to detect important interaction sites or hotspots without involving actual ligands. In this way, a protein-derived pharmacophore is obtained and defines energetically favorable binding site locations for docked compounds. A currently common technique for obtaining these hotspots is to run molecular dynamics simulations with small probes (see **Mixed Solvents** section). The hotspots can then be used to adjust the docking protocol, e.g., by adding a restriction towards the formation of a given protein-probe interaction. Recently, Arcon et al. showed that determination of water and/or ethanol sites derived from molecular dynamics simulations in mixed solvents allowed identification of over 79% of all protein-ligand interactions, especially those that were most important for the binding [54]. They also stated how this knowledge could be used to improve docking. On the other hand, a ligand-derived bias extracts the information from the known ligands for a particular protein target, for example, a particular substructure such as the core of a congeneric series. Several protein-ligand complex structures are available and the conserved interactions of the co-crystallized ligands (ligand-derived pharmacophore) can be inferred and used to improve docking accuracy [97,98].

The improved performance of knowledge-based biased docking is highlighted by the different options available in the most common docking programs. For example, Glide [99] and GOLD [100] allow hydrogen bonds and substructure-based constraints, while Glide also permits metal restraints to enforce coordination geometries. On the other hand, rDock [94] and MOE [101] are able to constrain generated poses to satisfy pharmacophores, and thus bias the results towards important interactions, and also perform knowledge-based template guided (or tethered) docking. DOCK6 [102] has a conformational search option to bias the sampling towards poses in accordance with a defined set of known ligand structures. AutoDock [92] and DOCK3 [103] were subjected, by us [29] and others [104,105], to implementations considering the energy accounted from water displacement through inhomogeneous solvation theory for guiding the docking. Lopez et al. have proposed a scheme to add a bias to AutoDock [29] that has been recently implemented for performing biased docking with AutoDock4 (AutoDock-Bias, in preparation Arcon et al., 2018). The versatile definition of the different types of biases in AutoDock-Bias accounts for all of the above cases. It allows guided docking towards pharmacophoric interactions in a straightforward way for hydrogen bond and hydrophobic/aromatic interactions. Furthermore, it allows researchers to get ideal interaction patterns for a specific protein structure, thus easily defining interactor locations. In addition, the capability of modifying any specific energy map and assigning any bias potential strength permits the precise localization of any desired atom (e.g., metal) or group (e.g., substructure core of a congeneric ligand series or for fragment growth) in a defined region space relative to the target protein. Finally, the specific energy map modification may also be used as an anchor for covalent docking studies. Since we addressed the problem of incorporating single target information, in the present discussion, we omitted potentials used for docking scoring functions [106,107,108] generally derived for diverse protein-ligand complexes.

In summary, mixed solvents simulations can lead to the identification of hot spots that can then be used in biased docking. The bias may affect the conformational search and/or scoring of the obtained poses. 

## 6. Conclusions and Perspectives 

Simulation of molecular dynamics in an explicit solvent are needed for accurate drug design. As the thermodynamics of the solvent reorganization upon drug binding is a key contribution to the complex formation free energy and thus to the ligand binding affinity. Therefore, accurate predictions have to consider the protein and the ligand embedded in the solvent, as part of a condensed state and have to account for a great number of configurational possibilities. On the other hand, explicit water MD allows studying the structure and dynamics of water molecules, and therefore the identification of water sites, that are relevant for their capacity to reveal key hydrophilic protein-ligand interaction sites. Water provides useful information for drug design, like guiding thermodynamic integration computations for compound optimization by allowing researchers to predict where it is favorable to grow the compound by displacing waters [109,110]. Another recent use of water molecules is to design specific inhibitors between a protein family, like the bromodomain proteins where structural water position determines drug selectivity [111]. The MD application of mixed solvents allows researchers to detect binding sites and quantify their potential to bind drug-like molecules. In turn, the identified hot spots can then be used as a bias in docking simulations to better identify drug candidates. 

Mixed solvent MD with a cosolvent of no more than 10 heavy atoms is feasible and as we have described in this review, can clearly contribute to drug design, but has not yet been fully exploited. With the advent of new web services and user-friendly software, good algorithms to analyze the simulations and faster computers, we expect to see an increase in the application of these techniques to a plethora of protein targets. Docking simulations have not increased accuracy for drug-protein conformational predictions in the last decade, but most probably will get better in the near future, with the increased use of knowledge-based algorithms. MDMix will also help to obtain more accurate binding free energy estimations, but much effort in the community is needed in order to derive new algorithms that are not only able to estimate the free energy contribution of drug-protein interactions, but also the free energy of protein and drug desolvation. 

## References

[1] Jorgensen W.L. (2004). The many roles of computation in drug discovery. Science.

[2] Sliwoski G., Kothiwale S., Meiler J., Lowe E.W. (2014). Computational methods in drug discovery. Pharmacol. Rev..

[3] Brown D.G., Boström J. (2018). Where Do Recent Small Molecule Clinical Development Candidates Come From?. J. Med. Chem..

[4] Bottaro S., Lindorff-Larsen K. (2018). Biophysical experiments and biomolecular simulations: A perfect match?. Science.

[5] Lazaridis T. (1998). Inhomogeneous Fluid Approach to Solvation Thermodynamics. 1. Theory. J. Phys. Chem. B.

[6] Pan A.C., Xu H., Palpant T., Shaw D.E. (2017). Quantitative Characterization of the Binding and Unbinding of Millimolar Drug Fragments with Molecular Dynamics Simulations. J. Chem. Theory Comput..

[7] Lee E.H., Hsin J., Sotomayor M., Comellas G., Schulten K. (2009). Discovery through the computational microscope. Structure.

[8] Goodford P.J. (1985). A computational procedure for determining energetically favorable binding sites on biologically important macromolecules. J. Med. Chem..

[9] Miranker A., Karplus M. (1991). Functionality maps of binding sites: A multiple copy simultaneous search method. Proteins.

[10] Brenke R., Kozakov D., Chuang G.-Y., Beglov D., Hall D., Landon M.R., Mattos C., Vajda S. (2009). Fragment-based identification of druggable “hot spots” of proteins using Fourier domain correlation techniques. Bioinformatics.

[11] Baum B., Muley L., Smolinski M., Heine A., Hangauer D., Klebe G. (2010). Non-additivity of functional group contributions in protein-ligand binding: A comprehensive study by crystallography and isothermal titration calorimetry. J. Mol. Biol..

[12] Biela A., Betz M., Heine A., Klebe G. (2012). Water Makes the Difference: Rearrangement of Water Solvation Layer Triggers Non-additivity of Functional Group Contributions in Protein--Ligand Binding. ChemMedChem..

[13] Eastman P., Swails J., Chodera J.D., McGibbon R.T., Zhao Y., Beauchamp K.A., Wang L.-P., Simmonett A.C., Harrigan M.P., Stern C.D. (2017). OpenMM 7: Rapid development of high performance algorithms for molecular dynamics. PLoS Comput. Biol..

[14] Kutzner C., Páll S., Fechner M., Esztermann A., de Groot B.L., Grubmüller H. (2015). Best bang for your buck: GPU nodes for GROMACS biomolecular simulations. J. Comput. Chem..

[15] Lee T.-S., Cerutti D.S., Mermelstein D., Lin C., LeGrand S., Giese T.J., Roitberg A., Case D.A., Walker R.C., York D.M. (2018). GPU-Accelerated Molecular Dynamics and Free Energy Methods in Amber18: Performance Enhancements and New Features. J. Chem. Inf. Model..

[16] Li Z., Lazaridis T. (2005). The effect of water displacement on binding thermodynamics: Concanavalin A. J. Phys. Chem. B.

[17] Englert L., Biela A., Zayed M., Heine A., Hangauer D., Klebe G. (2010). Displacement of disordered water molecules from hydrophobic pocket creates enthalpic signature: Binding of phosphonamidate to the S1′-pocket of thermolysin. BBA—Gen. Subj..

[18] Michel J., Tirado-Rives J., Jorgensen W.L. (2009). Energetics of displacing water molecules from protein binding sites: Consequences for ligand optimization. J. Am. Chem. Soc..

[19] García-Sosa A.T., Mancera R.L., Dean P.M. (2003). WaterScore: A novel method for distinguishing between bound and displaceable water molecules in the crystal structure of the binding site of protein-ligand complexes. J. Mol. Model..

[20] Crawford T.D., Tsui V., Flynn E.M., Wang S., Taylor A.M., Côté A., Audia J.E., Beresini M.H., Burdick D.J., Cummings R. (2016). Diving into the Water: Inducible Binding Conformations for BRD4, TAF1(2), BRD9, and CECR2 Bromodomains. J. Med. Chem..

[21] Ladbury J.E. (1996). Just add water! The effect of water on the specificity of protein-ligand binding sites and its potential application to drug design. Chem. Biol..

[22] Poornima C.S., Dean P.M. (1995). Hydration in drug design. 1. Multiple hydrogen-bonding features of water molecules in mediating protein-ligand interactions. J. Comput. Aided Mol. Des..

[23] Levinson N.M., Boxer S.G. (2014). A conserved water-mediated hydrogen bond network defines bosutinib’s kinase selectivity. Nat. Chem. Biol..

[24] García-Sosa A.T. (2013). Hydration properties of ligands and drugs in protein binding sites: Tightly-bound, bridging water molecules and their effects and consequences on molecular design strategies. J. Chem. Inf. Model..

[25] Sridhar A., Ross G.A., Biggin P.C. (2017). Waterdock 2.0: Water placement prediction for Holo-structures with a pymol plugin. PLoS ONE.

[26] Bissantz C., Kuhn B., Stahl M. (2010). A Medicinal Chemist’s Guide to Molecular Interactions. J. Med. Chem..

[27] Wiesner S., Kurian E., Prendergast F.G., Halle B. (1999). Water molecules in the binding cavity of intestinal fatty acid binding protein: Dynamic characterization by water 17O and 2H magnetic relaxation dispersion. J. Mol. Biol..

[28] Gauto D.F., Di Lella S., Guardia C.M.A., Estrin D.A., Martí M.A. (2009). Carbohydrate-binding proteins: Dissecting ligand structures through solvent environment occupancy. J. Phys. Chem. B.

[29] López E.D., Arcon J.P., Gauto D.F., Petruk A.A., Modenutti C.P., Dumas V.G., Marti M.A., Turjanski A.G. (2015). WATCLUST: A tool for improving the design of drugs based on protein-water interactions. Bioinformatics.

[30] Klibanov A.M. (2001). Improving enzymes by using them in organic solvents. Nature.

[31] Halling P.J. (2004). What can we learn by studying enzymes in non-aqueous media?. Philos. Trans. R. Soc. Lond. B Biol. Sci..

[32] Allen K.N., Bellamacina C.R., Ding X., Jeffery C.J., Mattos C., Petsko G.A., Ringe D. (1996). An Experimental Approach to Mapping the Binding Surfaces of Crystalline Proteins. J. Phys. Chem..

[33] Liepinsh E., Otting G. (1997). Organic solvents identify specific ligand binding sites on protein surfaces. Nat. Biotechnol..

[34] English A.C., Done S.H., Caves L.S., Groom C.R., Hubbard R.E. (1999). Locating interaction sites on proteins: The crystal structure of thermolysin soaked in 2% to 100% isopropanol. Proteins.

[35] English A.C., Groom C.R., Hubbard R.E. (2001). Experimental and computational mapping of the binding surface of a crystalline protein. Protein Eng..

[36] Clackson T., Wells J.A. (1995). A hot spot of binding energy in a hormone-receptor interface. Science.

[37] Caflisch A. (1996). Computational combinatorial ligand design: Application to human alpha-thrombin. J. Comput. Aided Mol. Des..

[38] Seco J., Luque F.J., Barril X. (2009). Binding site detection and druggability index from first principles. J. Med. Chem..

[39] Vukovic S., Huggins D.J. (2018). Quantitative metrics for drug-target ligandability. Drug Discov. Today.

[40] Barril X. (2013). Druggability predictions: Methods, limitations, and applications. Wiley Interdiscip. Rev. Comput. Mol. Sci..

[41] Guvench O., MacKerell A.D. (2009). Computational fragment-based binding site identification by ligand competitive saturation. PLoS Comput. Biol..

[42] Bakan A., Nevins N., Lakdawala A.S., Bahar I. (2012). Druggability Assessment of Allosteric Proteins by Dynamics Simulations in the Presence of Probe Molecules. J. Chem. Theory Comput..

[43] Ghanakota P., Carlson H.A. (2016). Moving Beyond Active-Site Detection: MixMD Applied to Allosteric Systems. J. Phys. Chem. B.

[44] Sayyed-Ahmad A., Gorfe A.A. (2017). Mixed-Probe Simulation and Probe-Derived Surface Topography Map Analysis for Ligand Binding Site Identification. J. Chem. Theory Comput..

[45] Yu W., Lakkaraju S.K., Raman E.P., Fang L., MacKerell A.D. (2015). Pharmacophore modeling using site-identification by ligand competitive saturation (SILCS) with multiple probe molecules. J. Chem. Inf. Model..

[46] Ghanakota P., van Vlijmen H., Sherman W., Beuming T. (2018). Large-Scale Validation of Mixed-Solvent Simulations to Assess Hotspots at Protein-Protein Interaction Interfaces. J. Chem. Inf. Model..

[47] Alvarez-Garcia D., Barril X. (2014). Molecular simulations with solvent competition quantify water displaceability and provide accurate interaction maps of protein binding sites. J. Med. Chem..

[48] Graham S.E., Smith R.D., Carlson H.A. (2018). Predicting Displaceable Water Sites Using Mixed-Solvent Molecular Dynamics. J. Chem. Inf. Model..

[49] Uehara S., Tanaka S. (2017). Cosolvent-Based Molecular Dynamics for Ensemble Docking: Practical Method for Generating Druggable Protein Conformations. J. Chem. Inf. Model..

[50] Oleinikovas V., Saladino G., Cossins B.P., Gervasio F.L. (2016). Understanding Cryptic Pocket Formation in Protein Targets by Enhanced Sampling Simulations. J. Am. Chem. Soc..

[51] Comitani F., Gervasio F.L. (2018). Exploring Cryptic Pockets Formation in Targets of Pharmaceutical Interest with SWISH. J. Chem. Theory Comput..

[52] Kimura S.R., Hu H.P., Ruvinsky A.M., Sherman W., Favia A.D. (2017). Deciphering Cryptic Binding Sites on Proteins by Mixed-Solvent Molecular Dynamics. J. Chem. Inf. Model..

[53] Raman E.P., Yu W., Guvench O., Mackerell A.D. (2011). Reproducing crystal binding modes of ligand functional groups using Site-Identification by Ligand Competitive Saturation (SILCS) simulations. J. Chem. Inf. Model..

[54] Arcon J.P., Defelipe L.A., Modenutti C.P., López E.D., Alvarez-Garcia D., Barril X., Turjanski A.G., Martí M.A. (2017). Molecular Dynamics in Mixed Solvents Reveals Protein-Ligand Interactions, Improves Docking, and Allows Accurate Binding Free Energy Predictions. J. Chem. Inf. Model..

[55] Ghanakota P., Carlson H.A. (2016). Driving Structure-Based Drug Discovery through Cosolvent Molecular Dynamics. J. Med. Chem..

[56] Pan A.C., Borhani D.W., Dror R.O., Shaw D.E. (2013). Molecular determinants of drug-receptor binding kinetics. Drug Discov. Today.

[57] Kuntz I.D., Chen K., Sharp K.A., Kollman P.A. (1999). The maximal affinity of ligands. Proc. Natl. Acad. Sci. USA.

[58] Huang D., Caflisch A. (2011). The free energy landscape of small molecule unbinding. PLoS Comput. Biol..

[59] Lexa K.W., Goh G.B., Carlson H.A. (2014). Parameter choice matters: Validating probe parameters for use in mixed-solvent simulations. J. Chem. Inf. Model..

[60] Foster T.J., MacKerell A.D., Guvench O. (2012). Balancing target flexibility and target denaturation in computational fragment-based inhibitor discovery. J. Comput. Chem..

[61] Alvarez-Garcia D., Barril X. (2014). Relationship between Protein Flexibility and Binding: Lessons for Structure-Based Drug Design. J. Chem. Theory Comput..

[62] Erlanson D.A., Fesik S.W., Hubbard R.E., Jahnke W., Jhoti H. (2016). Twenty years on: The impact of fragments on drug discovery. Nat. Rev. Drug Discov..

[63] Chen Y., Shoichet B.K. (2009). Molecular docking and ligand specificity in fragment-based inhibitor discovery. Nat. Chem. Biol..

[64] Giannetti A.M., Shoichet B.K. (2009). Docking for fragment inhibitors of AmpC β-lactamase. Proc. Natl. Acad. Sci. USA.

[65] Zhao H., Gartenmann L., Dong J., Spiliotopoulos D., Caflisch A. (2014). Discovery of BRD4 bromodomain inhibitors by fragment-based high-throughput docking. Bioorg. Med. Chem. Lett..

[66] Spiliotopoulos D., Zhu J., Wamhoff E.-C., Deerain N., Marchand J.-R., Aretz J., Rademacher C., Caflisch A. (2017). Virtual screen to NMR (VS2NMR): Discovery of fragment hits for the CBP bromodomain. Bioorg. Med. Chem. Lett..

[67] Vass M., Agai-Csongor E., Horti F., Keserű G.M. (2014). Multiple fragment docking and linking in primary and secondary pockets of dopamine receptors. ACS Med. Chem. Lett..

[68] Marchand J.-R., Dalle Vedove A., Lolli G., Caflisch A. (2017). Discovery of Inhibitors of Four Bromodomains by Fragment-Anchored Ligand Docking. J. Chem. Inf. Model..

[69] Jubb H., Blundell T.L., Ascher D.B. (2015). Flexibility and small pockets at protein-protein interfaces: New insights into druggability. Prog. Biophys. Mol. Biol..

[70] Chipot C., Pohorille A. (2007). Free Energy Calculations: Theory and Applications in Chemistry and Biology.

[71] Chen D., Ranganathan A., IJzerman A.P., Siegal G., Carlsson J. (2013). Complementarity between in silico and biophysical screening approaches in fragment-based lead discovery against the A(2A) adenosine receptor. J. Chem. Inf. Model..

[72] Steinbrecher T.B., Dahlgren M., Cappel D., Lin T., Wang L., Krilov G., Abel R., Friesner R., Sherman W. (2015). Accurate Binding Free Energy Predictions in Fragment Optimization. J. Chem. Inf. Model..

[73] Jiang W., Roux B. (2010). Free Energy Perturbation Hamiltonian Replica-Exchange Molecular Dynamics (FEP/H-REMD) for Absolute Ligand Binding Free Energy Calculations. J. Chem. Theory Comput..

[74] Aldeghi M., Heifetz A., Bodkin M.J., Knapp S., Biggin P.C. (2016). Accurate calculation of the absolute free energy of binding for drug molecules. Chem. Sci..

[75] Lin Y.-L., Meng Y., Jiang W., Roux B. (2013). Explaining why Gleevec is a specific and potent inhibitor of Abl kinase. Proc. Natl. Acad. Sci. USA.

[76] Dror R.O., Pan A.C., Arlow D.H., Borhani D.W., Maragakis P., Shan Y., Xu H., Shaw D.E. (2011). Pathway and mechanism of drug binding to G-protein-coupled receptors. Proc. Natl. Acad. Sci. USA.

[77] Mondal J., Friesner R.A., Berne B.J. (2014). Role of Desolvation in Thermodynamics and Kinetics of Ligand Binding to a Kinase. J. Chem. Theory Comput..

[78] Tiwary P., Mondal J., Berne B.J. (2017). How and when does an anticancer drug leave its binding site?. Sci. Adv..

[79] Lotz S.D., Dickson A. (2018). Unbiased Molecular Dynamics of 11 min Timescale Drug Unbinding Reveals Transition State Stabilizing Interactions. J. Am. Chem. Soc..

[80] Plattner N., Noé F. (2015). Protein conformational plasticity and complex ligand-binding kinetics explored by atomistic simulations and Markov models. Nat. Commun..

[81] Bisignano P., Doerr S., Harvey M.J., Favia A.D., Cavalli A., De Fabritiis G. (2014). Kinetic characterization of fragment binding in AmpC β-lactamase by high-throughput molecular simulations. J. Chem. Inf. Model..

[82] Ferruz N., Harvey M.J., Mestres J., De Fabritiis G. (2015). Insights from Fragment Hit Binding Assays by Molecular Simulations. J. Chem. Inf. Model..

[83] Buch I., Giorgino T., De Fabritiis G. (2011). Complete reconstruction of an enzyme-inhibitor binding process by molecular dynamics simulations. Proc. Natl. Acad. Sci. USA.

[84] Rathi P.C., Ludlow R.F., Hall R.J., Murray C.W., Mortenson P.N., Verdonk M.L. (2017). Predicting “Hot” and “Warm” Spots for Fragment Binding. J. Med. Chem..

[85] Martinez-Rosell G., Harvey M.J., De Fabritiis G. (2018). Molecular-Simulation-Driven Fragment Screening for the Discovery of New CXCL12 Inhibitors. J. Chem. Inf. Model..

[86] Ludlow R.F., Verdonk M.L., Saini H.K., Tickle I.J., Jhoti H. (2015). Detection of secondary binding sites in proteins using fragment screening. Proc. Natl. Acad. Sci. USA.

[87] Shan Y., Kim E.T., Eastwood M.P., Dror R.O., Seeliger M.A., Shaw D.E. (2011). How does a drug molecule find its target binding site?. J. Am. Chem. Soc..

[88] Colizzi F., Perozzo R., Scapozza L., Recanatini M., Cavalli A. (2010). Single-molecule pulling simulations can discern active from inactive enzyme inhibitors. J. Am. Chem. Soc..

[89] Gioia D., Bertazzo M., Recanatini M., Masetti M., Cavalli A. (2017). Dynamic Docking: A Paradigm Shift in Computational Drug Discovery. Molecules.

[90] Casasnovas R., Limongelli V., Tiwary P., Carloni P., Parrinello M. (2017). Unbinding Kinetics of a p38 MAP Kinase Type II Inhibitor from Metadynamics Simulations. J. Am. Chem. Soc..

[91] Ruiz-Carmona S., Schmidtke P., Luque F.J., Baker L., Matassova N., Davis B., Roughley S., Murray J., Hubbard R., Barril X. (2017). Dynamic undocking and the quasi-bound state as tools for drug discovery. Nat. Chem..

[92] Morris G.M., Huey R., Lindstrom W., Sanner M.F., Belew R.K., Goodsell D.S., Olson A.J. (2009). AutoDock4 and AutoDockTools4: Automated docking with selective receptor flexibility. J. Comput. Chem..

[93] Forli S., Huey R., Pique M.E., Sanner M.F., Goodsell D.S., Olson A.J. (2016). Computational protein-ligand docking and virtual drug screening with the AutoDock suite. Nat. Protoc..

[94] Ruiz-Carmona S., Alvarez-Garcia D., Foloppe N., Garmendia-Doval A.B., Juhos S., Schmidtke P., Barril X., Hubbard R.E., Morley S.D. (2014). rDock: A fast, versatile and open source program for docking ligands to proteins and nucleic acids. PLoS Comput. Biol..

[95] Sousa S.F., Ribeiro A.J.M., Coimbra J.T.S., Neves R.P.P., Martins S.A., Moorthy N.S., Fernandes P.A., Ramos M.J. (2013). Protein-Ligand Docking in the New Millennium—A Retrospective of 10 Years in the Field. Curr. Med. Chem..

[96] Cleves A.E., Jain A.N. (2015). Knowledge-guided docking: Accurate prospective prediction of bound configurations of novel ligands using Surflex-Dock. J. Comput. Aided Mol. Des..

[97] Hu B., Lill M.A. (2014). PharmDock: A pharmacophore-based docking program. J. Cheminform..

[98] Perryman A.L., Santiago D.N., Forli S., Martins D.S., Olson A.J. (2014). Virtual screening with AutoDock Vina and the common pharmacophore engine of a low diversity library of fragments and hits against the three allosteric sites of HIV integrase: Participation in the SAMPL4 protein-ligand binding challenge. J. Comput. Aided Mol. Des..

[99] Friesner R.A., Banks J.L., Murphy R.B., Halgren T.A., Klicic J.J., Mainz D.T., Repasky M.P., Knoll E.H., Shelley M., Perry J.K. (2004). Glide: A new approach for rapid, accurate docking and scoring. 1. Method and assessment of docking accuracy. J. Med. Chem..

[100] Jones G., Willett P., Glen R.C., Leach A.R., Taylor R. (1997). Development and validation of a genetic algorithm for flexible docking. J. Mol. Biol..

[101] Corbeil C.R., Williams C.I., Labute P. (2012). Variability in docking success rates due to dataset preparation. J. Comput. Aided Mol. Des..

[102] Allen W.J., Balius T.E., Mukherjee S., Brozell S.R., Moustakas D.T., Lang P.T., Case D.A., Kuntz I.D., Rizzo R.C. (2015). DOCK 6: Impact of new features and current docking performance. J. Comput. Chem..

[103] Coleman R.G., Carchia M., Sterling T., Irwin J.J., Shoichet B.K. (2013). Ligand pose and orientational sampling in molecular docking. PLoS ONE.

[104] Balius T.E., Fischer M., Stein R.M., Adler T.B., Nguyen C.N., Cruz A., Gilson M.K., Kurtzman T., Shoichet B.K. (2017). Testing inhomogeneous solvation theory in structure-based ligand discovery. Proc. Natl. Acad. Sci. USA.

[105] Uehara S., Tanaka S. (2016). AutoDock-GIST: Incorporating Thermodynamics of Active-Site Water into Scoring Function for Accurate Protein-Ligand Docking. Molecules.

[106] Gohlke H., Hendlich M., Klebe G. (2000). Knowledge-based scoring function to predict protein-ligand interactions. J. Mol. Biol..

[107] Muegge I., Martin Y.C. (1999). A general and fast scoring function for protein-ligand interactions: A simplified potential approach. J. Med. Chem..

[108] Zheng Z., Merz K.M. (2013). Development of the Knowledge-Based and Empirical Combined Scoring Algorithm (KECSA) To Score Protein–Ligand Interactions. J. Chem. Inf. Model..

[109] Wang L., Wu Y., Deng Y., Kim B., Pierce L., Krilov G., Lupyan D., Robinson S., Dahlgren M.K., Greenwood J. (2015). Accurate and reliable prediction of relative ligand binding potency in prospective drug discovery by way of a modern free-energy calculation protocol and force field. J. Am. Chem. Soc..

[110] García-Sosa A.T., Mancera R.L. (2010). Free Energy Calculations of Mutations Involving a Tightly Bound Water Molecule and Ligand Substitutions in a Ligand-Protein Complex. Mol. Inform..

[111] Aldeghi M., Ross G.A., Bodkin M.J., Essex J.W., Knapp S., Biggin P.C. (2018). Large-scale analysis of water stability in bromodomain binding pockets with grand canonical Monte Carlo. Commun. Chem..

